# A general role for medial prefrontal cortex in event prediction

**DOI:** 10.3389/fncom.2014.00069

**Published:** 2014-07-11

**Authors:** William H. Alexander, Joshua W. Brown

**Affiliations:** ^1^Department of Experimental Psychology, Ghent UniversityGent, Belgium; ^2^Department of Psychological and Brain Sciences, Indiana University, BloomingtonBloomington, IN, USA

**Keywords:** medial prefrontal cortex, anterior cingulate, cognitive control, attention, reinforcement learning

## Abstract

A recent computational neural model of medial prefrontal cortex (mPFC), namely the predicted response-outcome (PRO) model (Alexander and Brown, 2011), suggests that mPFC learns to predict the outcomes of actions. The model accounted for a wide range of data on the mPFC. Nevertheless, numerous recent findings suggest that mPFC may signal predictions and prediction errors even when the predicted outcomes are not contingent on prior actions. Here we show that the existing PRO model can learn to predict outcomes in a general sense, and not only when the outcomes are contingent on actions. A series of simulations show how this generalized PRO model can account for an even broader range of findings in the mPFC, including human ERP, fMRI, and macaque single-unit data. The results suggest that the mPFC learns to predict salient events in general and provides a theoretical framework that links mPFC function to model-based reinforcement learning, Bayesian learning, and theories of cognitive control.

## Introduction

Medial prefrontal cortex (mPFC), especially dorsal anterior cingulate cortex (ACC) has been repeatedly and extensively implicated in processing and monitoring behavior and action (Falkenstein et al., 1991; Carter et al., 1998; Shima and Tanji, 1998; Botvinick et al., 2001; Holroyd and Coles, 2002; Behrens et al., 2007; Matsumoto et al., 2007; Rudebeck et al., 2008). A new unified model of the mPFC, the *predicted response-outcome* (PRO) model (Alexander and Brown, 2011), proposes that mPFC learns predictions of future outcomes, and signals unexpected non-occurrences of predicted outcomes. The model comprehensively accounts for a range of results observed in mPFC (including from fMRI, EEG, and single-unit neurophysiology) in the context of cognitive control, including effects of error, conflict, error likelihood, and several others.

While earlier simulations of the PRO model focused on the role of mPFC in predicting the outcomes of actions, the mPFC is also engaged in tasks without a significant behavioral component, or when a specific motor command is neither planned nor executed (Büchel et al., 2002; Chandrasekhar et al., 2008), in processing novel stimuli (Dien et al., 2003; Crottaz-Herbette and Menon, 2006), in predicting task-related stimuli that cue future behavior but require no immediate response (Koyama et al., 2001; Aarts et al., 2008; Aarts and Roelofs, 2010), and in response to painful stimuli (Büchel et al., 2002; Chandrasekhar et al., 2008). These findings suggest a role for mPFC in deploying attention (Bryden et al., 2011; Vachon et al., 2012) and processing novelty or salience (Downar et al., 2002; Litt et al., 2011; Wessel et al., 2012).

These findings present a significant challenge to accounts of mPFC function that emphasize its role in the regulation and correction of behavior alone. Furthermore, theories regarding mPFC function will necessarily be incomplete so long as findings regarding the role of mPFC in processing stimuli remain unexplained. One possibility is that stimulus-related activity in mPFC reflects a separate, independent function of mPFC which operates concurrently with mPFC involvement in control of behavior. A second option is that these findings are a product of the same mechanisms that produce effects in mPFC related to action and outcome.

Can the same principle that informed the PRO model, prediction of likely outcomes and detection of unexpected non-occurrence, be deployed to explain mPFC activity related to task-related cues? In order to answer this question, we first (re)consider what we mean by “outcome”. In the original PRO model, outcomes were conceived as events, usually reflecting performance-related feedback, occurring at the end of a trial. After the model was presented with an outcome, all learning within the model ceased and all activity was set to 0 in order to prepare the model for the next trial.

In reality, however, a person’s experience is not divided into discrete trials in this fashion. Even in the highly-constrained reality of a behavioral experiment, trials are followed by still more trials, each identical to the last modulo experimental manipulations. Each time an “outcome” is observed by a subject, it is reliably followed by a stimulus indicating the onset of a new trial, which is itself followed by another outcome, *ad infinitum* (or at least until the experimenter allows the subject to leave). From this perspective, the distinction between an outcome and a stimulus becomes ambiguous, with the difference seeming to rest on experimenter *fiat*.

**Figure 1:**
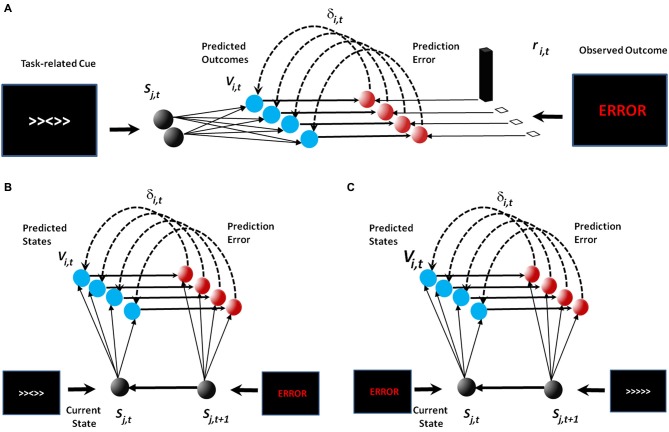
**Model schematics**. In the original publication of the PRO model **(A)**, the model learned predictions of future outcomes (e.g., error or correct feedback) based on task-related cues such as those observed in the Eriksen flanker task. In our extension to the PRO model (**B** and **C**), the model continues to learn the association between task-related cues and feeback **(B)**. Task-related feedback then acts as a stimulus in its own right in order to learn associations between feedback and future task-related cues **(C)**.

With this in mind, we propose a modest extension to the original PRO model (Figure 1). Namely, in the extended PRO model, we regard stimuli and their associated outcomes as generic *events*, where events are considered to be any salient sensory input that can be associated with subsequent events, and may itself be predicted by previous events. It is essential to note from the outset that *this extension is a conceptual expansion only, and the extended PRO model below is identical to the original PRO model, including all the same equations and parameters*. With this simple conceptual extension, we are able to demonstrate how the PRO model, in addition to accounting for mPFC activity associated with response monitoring, can reproduce a range of effects observed in mPFC and related primarily to processing sensory stimuli from fMRI, EEG, and single-unit neurophysiological studies. These findings provide additional evidence that the hypothesis underlying the PRO model, that mPFC is involved in prediction and detecting discrepancies, is the most comprehensive account of mPFC function to date.

## Methods

The PRO model was developed to account for mPFC activity related to the prediction of response-outcome conjunctions, and signaling unexpected deviations from expected outcomes. In our extended implementation of the PRO model, we generalize these two basic functions of the PRO model to include prediction of any salient sensory event (including outcomes), as well signaling deviations from expected events. In order to describe our implementation of the extended model, we first review relevant equations from the original model, and then show how these equations have been updated to generalize the events they represent.

## PRO model

In order to explain effects observed in mPFC related to the prediction and observation of outcomes following a behavioral response, the original PRO model is based on standard reinforcement learning (RL) models, especially temporal difference (TD) learning (Sutton, 1988), that have been extended in the following ways. First, in typical formulations, RL models learn a scalar prediction of the discounted value of the current state. In contrast, the PRO model learns predictions of multiple possible outcomes, regardless of their affective valence, using a vector-valued error signal. Activity in the PRO model therefore reflects a temporally discounted prediction of various outcomes in proportion their probability of occurrence. Second, mPFC effects related to error are explained as “negative surprise”, a value which reflects the aggregate of outcome predictions generated by the model minus observed outcomes. The PRO model represents time as a tapped-delay line in which each unit reflects the amount of time elapsed since the presentation of a stimulus. Each iteration of the model was interpreted as lasting 10 ms.

Formally, predictions in the model are computed as:

(1) $$P_{i,t}=\sum_{j,k}S_{jk,t}\times W_{ijk,t}$$

where *S* is the tapped-delay representation of a stimulus, *W* are learned prediction weights associating stimuli with possible outcomes, *P*, and *i,j* and *k* index outcomes, tapped-delay units, and stimulus identity, respectively. Weights are updated according to:

(2) $$W_{ijk,t+1}=W_{ijk,t}+\alpha \delta_{i,t}{\bar{S}}_{jk}$$

where *α* is a learning rate parameter. *W* is further constrained by *W > 0*. $\bar{S}$ is an eligibility trace computed as:

(3) $${\bar{S}}_{jk,t+1}=S_{jk,t}+0.95{\bar{S}}_{jk,t}$$

Finally, δ is a TD error:

(4) $$\delta_{i,t}=O_{i,t}+\gamma P_{i,t+1}-P_{i,t}$$

where *O* is the outcome *i* observed on the current model iteration *t*, and *γ* is a temporal discount factor (*γ* = 0.95).

## Extended model

As described above, the central premise underlying our extended implementation of the PRO model is that outcomes and the stimuli which precede them can be regarded as generic events, by which we mean any salient information (i.e., experimental variables) a subject may encounter in the course of an experiment, up to and including information that may not pertain to the experimental task as such but merely signals the onset of a new trial (e.g., fixation points). Accordingly, the relevant equations given above are rewritten as:

(5) $$P_{i,t}=\sum_{j,k}E_{jk,t}\times W_{ijk,t}$$

(6) $$W_{ijk,t+1}=W_{ijk,t}+\alpha \delta_{i,t}{\bar{E}}_{jk}$$

(7) $${\bar{E}}_{jk,t+1}=E_{jk,t}+0.95{\bar{E}}_{jk,t}$$

(8) $$\delta_{i,t}=E_{i,t}+\gamma P_{i,t+1}-P_{i,t}$$

These equations are identical with Eqs. 1–4, with the exception that all instances of *S* and *O* are now replaced by *E*, reflecting the more general role of both stimuli and outcomes as events that can be predicted as well as serve as the basis for predicting future events. In order to accommodate learning predictions about the relationship between events, broadly construed, the model was further altered by allowing learning to occur even after the conclusion of a trial. Finally, activity in the model was computed as “negative surprise”:

(9) $$\omega_{t}^{N}={\sum_{i}\left\lfloor P_{i,t}-E_{i,t}\right\rfloor }^{+}$$

reflecting the unexpected non-occurrence of a predicted event. Except for simulation 6 (discussed below), this measure of model activity is used in all simulations.

In addition to these four core equations, the original PRO model incorporated mechanisms by which the model was able to interact with simulated cognitive control tasks. These mechanisms remain unchanged, and the parameters used for previous simulations are the same as previously reported (Alexander and Brown, 2011). These parameters were derived from model fits to behavioral data from a previously reported study (Brown and Braver, 2005). Model parameters were not altered from one simulation to the next. For simulations in which an event was not associated with a particular behavior (e.g., experiments in which certain stimuli do not require a response), stimulus-response weights in the model were set to 0.

## Simulations

Unless otherwise note, simulated experiments included 10 individual simulations, each corresponding to a single subject, of the PRO model in the tasks described below. In each task, or in each experimental condition within each task, the model was presented with 300 trials. At the beginning of each individual simulation, adjustable model weights were set to 0. Because trials for each task were selected randomly, and because responses were influenced both by learned and static weights as well as by an additional noise component, the development of activity in the model varied from one individual simulation to the next. In our simulations, we did not simulate variability in inter-trial or inter-stimulus intervals due to the dependence of the model on consistent timing of events to converge (resulting from its formulation based on TD learning).

### Simulation 1: Frequent vs. infrequent trials

Effects of trial frequency on model activity were simulated using an Eriksen flanker task (Eriksen, 1995) in two separate simulated experiments in which the frequency of trial types (congruent and incongruent) was manipulated. In the frequent condition for both experiments, frequent trials were observed approximately 75% of the time, while infrequent trials were approximately 25% of all trials. A total of eight events were modeled: left and right target cues, left and right flanker cues, as well as the four possible response-outcome conjunctions (left/error, right/error, left/correct, right/correct). Model activity was averaged over the first 20 model iterations following the onset of the target and flanker cues.

### Simulation 2: Item-specific vs. global control

The model was run in three separate simulated experiments using a version of the Stroop task (Stroop, 1935) in which the frequency of congruent vs. incongruent trials was manipulated both at a global level, as well as at the level of individual stimuli as in Blais and Bunge (2010). In each experiment, two classes of stimuli were used. In each stimulus class, two specific colors could be combined to generate Stroop stimuli. For example, one stimulus class might include the colors red and green used to generate incongruent and congruent stimuli—the word “red” displayed in green font, or vice versa (incongruent trials), or the word “red” (or green) displayed in red (or green) font (congruent trials), while the 2nd stimulus class would generate stimuli using two different colors (e.g., yellow and blue). In each experiment, both the global probability of observing an incongruent trial (regardless of stimulus class), as well as the item-specific (class-dependent) probability of observing an incongruent trial were manipulated. In the 1st experiment, the global probability of observing an incongruent trial was 0.3, while the item-specific probability was 0.1 and 0.5 for the two stimulus class. In the second experiment, both the global and item-specific probabilities of an incongruent trial were 0.5. Finally, in experiment 3, the global probability was 0.7 while the item-specific probabilities were 0.5 and 0.9 for the two stimulus classes. In each simulated experiment, a total of eight events were modeled: one event for each color word, and one for each font color, as well as four possible response-outcome conjunctions (Color1/Error, Color2/Error, Color1/Correct, Color2/Correct). For each experiment, the model was simulated for 200 trials, and model activity was averaged over the first 20 iterations following presentation of the stimulus.

### Simulation 3: Mismatch negativity

The mismatch negativity (MMN) was simulated as a punctuate stimulus presented to the model that repeated every 30 model iterations (300 ms). Since no response was required by the model, components of the PRO model related to response generation were lesioned by setting all weights for connections projecting to and from those components to 0. The model was trained on the repeating stimulus for 200 repetitions, following which single trials were simulated in which the stimulus was withheld following a number of repetitions (1–7). Model activity for all trials involving a withheld stimulus was averaged together regardless of the number of stimulus repetitions observed prior to the withheld stimulus, and activity for was recorded for the 40 iterations prior to the usual presentation time of the stimulus to 20 iterations after the usual presentation. Model activity for non-mismatch trials was averaged over all trials in which a stimulus was presented as expected, and activity was recorded as for mismatch trials.

### Simulation 4: Informative vs. uninformative cues

The task used by Aarts et al. (2008) was an arrow-word version of the Stroop task in which subjects were presented with both a word and visual cue indicating the direction in which they should respond (e.g., the word “right” printed within an arrow pointing left). On congruent trials, both the word and the visual cue indicated the same direction, while on incongruent trials, the word and visual cue indicated opposite responses. Prior to the onset of the task itself, subjects were presented one of three possible cues, each of which indicated whether the upcoming task would involve an incongruent trial (approximately 1/3 of all trials), a congruent trial (approximately 1/3 of all trials), or providing no information as to the nature of the trial (approximately 1/3 of all trials). A total of 11 events were modeled: 1 for each of the cue conditions (informed/congruent, informed/incongruent, uninformative), 3 events for task stimuli (1 for the central target stimulus, and 1 each for congruent and incongruent flankers) and 4 for the possible response-outcome conjunctions (left/error, left/correct, right/error, right/correct). Model activity was averaged over the 20 iterations following cue presentation for cue-related effects, and averaged over the 20 iterations following presentation of the trial (and preceding the model response or feedback) for target-related effects.

### Simulation 5: Bayesian surprise

In the stop signal task, subjects are presented with a cue indicating that a response is to be made. On a subset of trials, the subjects are subsequently presented with a second cue indicating that the subject should cancel the response to the first cue. We simulated the PRO model performing the stop signal task with the same frequency of go vs. stop trials reported in Ide et al. (2013) (75% and 25%, respectively). Model activity was averaged over the 20 model iterations following the presentation of a Stop cue. For each trial, the probability of observing a stop trial was calculated as proportion of stop trials over the previous ten trials. High and low probability trials were classified by a median split of the estimated probabilities of all trials experienced by the model. Seven events were modeled: 1 for the fixation point presented at the beginning of each trial, 1 each for the go and stop signals, and 4 for the possible response-outcome conjunctions (Go/Correct, Go/Error, Stop/Correct, and Stop/Error).

### Simulation 6: Single-unit activity

In the expect reward task (Sallet et al., 2007) conducted with monkeys, the animal was presented with a cue indicating the magnitude of a reward that would be delivered following a subsequent presentation of the same cue. Reward magnitudes could be either small, medium or large. On a subset of trials in the large and small magnitude conditions, the cue for the opposite reward (small instead of large, large instead of small) was presented following the initial cue. We simulated the PRO model on 200 trials of the expect reward task. A total of 10 events were modeled: 1 event for the starting position presented at the beginning of the trial, 3 events represented the reward magnitude cues during the Cue phase of the trial, 3 events represented the reward cues presented during the Go phase of each trial, and 3 events rewarded the reward received (small, medium, or large). Note that the activity of reward events was binary, and was intended to simulate the identity of the reward rather than its salience or value. This is consistent with the theory underlying the PRO model that states that mPFC learns the likely outcomes of actions rather than the value of those outcomes. Activity for cue related activity was averaged over 20 iterations following the presentation of the first cue and, separately, following the presentation of the second cue. Since we sought to account for single-unit activity, the activity of single units in the model was computed as in Eq. 9, but the results were not summed.

## Results

In previously published simulations (Alexander and Brown, 2011), we selected tasks on which to test the PRO model based on their potential to highlight a key strength of the model. Namely, we showed how the straightforward intuition underlying the model, that mPFC predicts future outcomes and signals deviations from expectations, can account for a wide range of data under a single, unifying framework. Specifically, we showed how the PRO model accounted for data from fMRI, EEG, and single-unit neurophysiology studies, while also showing how Bayesian accounts of mPFC activity could be reconciled with RL formulations. At the same time, we demonstrated that the PRO model, beyond capturing effects also accounted for by other models of mPFC (e.g., Botvinick et al., 2001; Holroyd and Coles, 2002; Brown and Braver, 2005), could additionally reproduce patterns of activity competing models could not (e.g., Amador et al., 2000; Amiez et al., 2006; Jessup et al., 2010).

Our goal in the present study is similar, in that we seek to demonstrate how, with a minimal amount of alteration, the PRO model may be extended to address results from the neuroscience literature showing mPFC involvement in the expectation and detection of stimuli. Accordingly, the data we have chosen to simulate include results from fMRI, EEG, and single-unit neurophysiology studies, as well as results implicating mPFC in Bayesian surprise.

### Simulation 1: Frequent vs. infrequent trials

**Figure 2:**
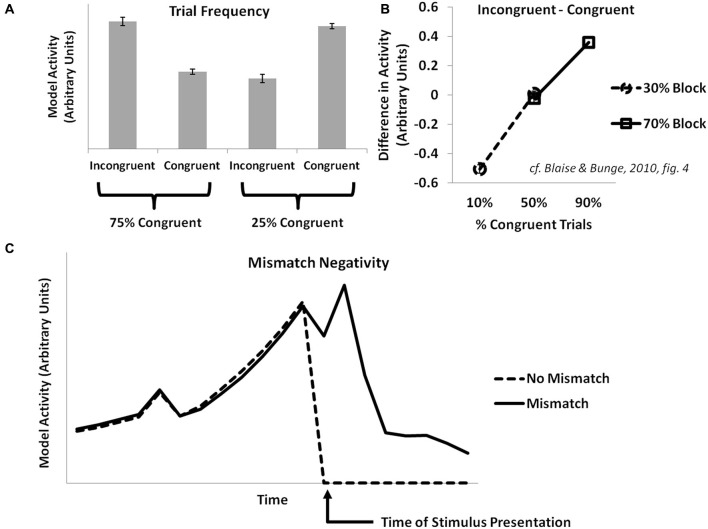
**Trial frequency, item-level control, and the mismatch negativity. (A)** Activity in the PRO model at the onset of a trial in the Eriksen flanker task reflects the overall frequency with which a particular trial type (congruent or incongruent) is observed. When mostly congruent trials are experienced, infrequent incongruent trials result in increased model activity relative to congruent trials, while the reverse holds true for conditions in which mostly incongruent trials are observed. **(B)** Activity in the model is proportional to the frequency with a particular trial type (e.g., incongruent or congruent) is observed with respect to a particular stimulus type (e.g., Stroop stimuli constructed using the color pair RED and GREEN vs. stimuli constructed using the color pair BLUE and YELLOW), and is not sensitive to the overall frequency of a trial type without regard for stimulus types. **(C)** Activity in the PRO model is greater following the surprise absence of a stimulus that commonly occurs as part of a sequence of stimuli (cf. Crottaz-Herbette and Menon, 2006).

Some fMRI and EEG studies manipulate the relative frequency of congruent vs. incongruent trials in common cognitive control tasks (e.g., the Eriksen flanker task or the Stroop task). They have observed an inverse correlation of conflict-related effects with the frequency of incongruent trials (Carter et al., 2000). The PRO model explains this as an increased prediction of the likelihood of an incongruent trial occurring in high-frequency incongruent conditions, with an attendant decrease in surprise when a predicted incongruent trial is experienced (Figure 2A). These studies also find that activity for infrequent incongruent trials is greater than for infrequent congruent trials when trials are matched for frequency. The PRO model captures this effect and explains it, as in previously published simulations, as the effect of multiple concurrent predictions for incongruent trials that proceed from the appearance of an incongruent stimulus.

### Simulation 2: Item-level vs. global control

The conflict model of ACC/mPFC suggests that cognitive control is proportional to the global statistics of a task; as the proportion of incongruent trials increases, so too does the overall need for top-down control to be deployed in order to successfully perform a task, with a resultant decrease in levels of conflict-related activity in ACC. However, both behavioral and fMRI studies (Bugg et al., 2008; Blais and Bunge, 2010) investigating this prediction have found that control appears to depend on the frequency of item-specific incongruent trials; particular stimuli associated with a higher proportion of incongruent trials appear to benefit more from adaptation effects relative to stimuli with a lower proportion of incongruent trials. Accordingly, since the PRO model learns predictions of likely events contingent on stimuli presented, simulated model activity at the onset of incongruent trials is inversely proportional to the overall item-specific frequency of incongruent trials (Figure 2B).

### Simulation 3: Mismatch negativity

The MMN ERP component is observed when, in the course of presentation of a predictable sequence of stimuli, a particular stimulus within that sequence is surprisingly altered (e.g., a high tone rather than a usual low tone) or withheld altogether. The MMN is most apparent in sensory cortices related to the stimulus modality, though EEG studies have also identified generators in frontal cortex, especially mPFC (Crottaz-Herbette and Menon, 2006) with an onset delayed compared to sensory cortex. The PRO model accounts for the MMN observed within mPFC as the surprising absence of a stimulus in a sequence whose occurrence was predicted by the previous stimulus (Figure 2C). Note that because activity in the PRO model derives entirely from the unexpected non-occurrence of an expected event, the model’s interpretation of the MMN remains the same regardless whether a predicted stimulus in a sequence is absent, or if a novel stimulus is inserted in its place (i.e., oddball paradigm). In both cases, the predicted event failed to occur.

### Simulation 4: Informative vs. uninformative cues

**Figure 3:**
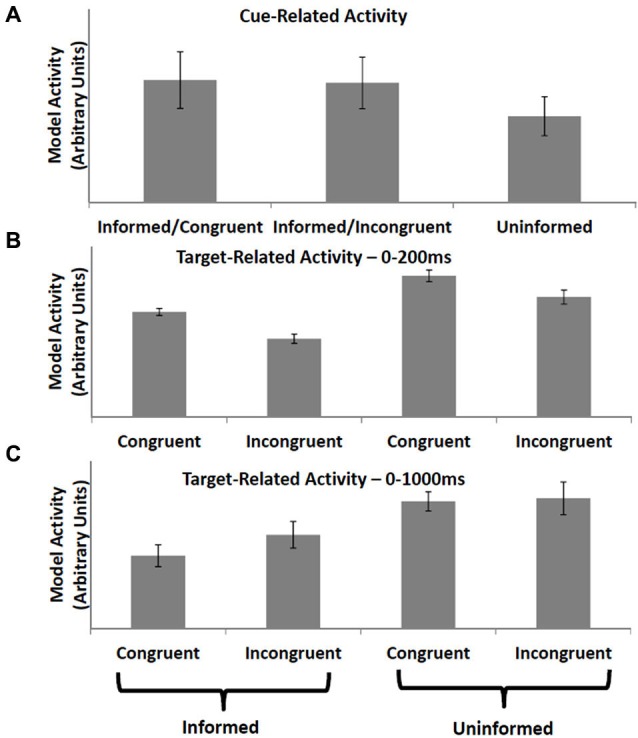
**Informative vs. uninformative cues**. **(A)** Activity in the PRO model is greater when a cue is presented indicating the type of trial (e.g., incongruent or congruent) that will be presented to the model in the near future, as compared to a cue that is uninformative (i.e., equal chance of either trial type). **(B)** Conversely, when a trial is presented, model activity is lower when the trial type has been previously cued compared to trials that have been preceded by an uninformative cue. **(C)** When model activity is recorded over a longer duration following trial presentation, reflecting the low temporal resolution of fMRI, trial-related activity for incongruent trials increases relative to high temporal resolution recording (frame B).

Aarts et al. (2008) observed increased activity in ACC following informative cues (cues which indicated whether the subject would subsequently perform a congruent or incongruent trial of a modified Stroop task) vs. uninformative cues. ACC activity at the time the cued task was presented was lower following informative cues relative to tasks occurring after uninformative cues, regardless of whether the trial itself was incongruent or congruent. The PRO model accounts for increased activity following an informative cue (Figure 3A) as the increased predictive activity related to the certain occurrence of either an incongruent or congruent trial vs. the weak activity following uninformative cues related to uncertain predictions regarding the nature of the next trial. Similarly, activity at the onset of the target task following an informative cue is reduced regardless of trial type (Figure 3B) since the model’s prediction corresponds with the observed event, while activity at trial-onset following uninformative cues reflects the unexpected non-occurrence of at least one of the model’s predictions. Note that although the PRO model captures the broad pattern observed in Aarts et al. (2008), the model reverses the direction of the effect observed at the onset of congruent tasks vs. incongruent task following uninformative cues. To explain this discrepancy between model predictions and empirical results, we note that our simulations sampled only a limited window of time following tonset of the task, equivalent to 200 ms of real time and far below the 2100 ms repetition time used by Aarts et al. to obtain their data. During this window, subjects were required to perform the task and monitor the outcomes of their behavioral responses. We therefore simulated the Aarts task again, this time using a window of 1000 ms following the onset of the target task, and find that the discrepancy between congruent and incongruent trials in the uninformed condition is eliminated (Figure 3C). In this simulation, all inter-stimulus and inter-trial intervals were identical to the initial simulation. In the model, increased activity to uninformed congruent trials (relative to uninformed incongruent trials) in the first 200 ms following task onset is due to stronger predictive activity related to the almost certain successful completion of the congruent task. At longer intervals, activity for uninformed incongruent trials is higher relative to uninformed congruent trials due to both the increased timed needed to perform an incongruent trial, as well as surprise signals related to both correct and incorrect performance. At the temporal resolution at which data can be measured via fMRI, these early and late components of the task are not separable. Our finding of differential model activity for incongruent vs. congruent trials during early periods following the onset of the target task is a novel prediction of the PRO model which may be tested using techniques with higher temporal resolution than standard fMRI allows.

### Simulation 5: Bayesian surprise

**Figure 4:**
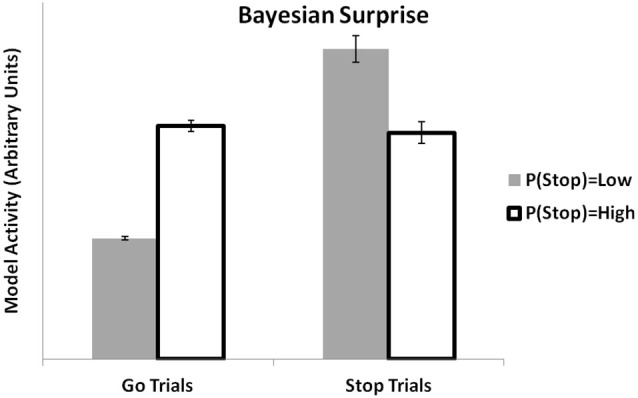
**Bayesian surprise**. The PRO model reproduces the pattern of activity observed in ACC based on local estimates of the likelihood of observing STOP or GO trials in a stop signal task. Activity at the onset of a GO trial is higher when the estimated likelihood of observing a STOP trial is high. Conversely, activity at the onset of a STOP trial is higher when the estimated likelihood of a STOP trial is believed to be low.

MPFC activity has been linked to computations related to Bayesian decision-making. In previous simulations, we showed how the PRO model might establish a link between mechanistic models of mPFC with more abstract Bayesian models by showing that it could reproduce effects of environmental volatility (Behrens et al., 2007) as estimated by a Bayesian algorithm. Recently, Ide et al. (2013) applied a Bayesian model (the Dynamic Belief Model (Yu et al., 2009)) to the analysis of fMRI data from a stop-signal task. The Dynamic Belief Model updates its estimation of the likelihood of observing a stop-signal trial based on the recent history of stop and go trials that have been observed. This estimation is used to calculate a Bayesian surprise signal, essentially the unsigned prediction error calculated as the absolute difference between the model’s estimation of the probability of a trial type and the actual trial type observed. The PRO model, which at its core is a model concerned with predicting likely events and signaling discrepancies between observed and actual events, accounts for the data in much the same way as reported earlier (Ide et al., 2013). When faced with a Stop trial, the activity of the PRO model is higher for situations in which recent trials have included only a few Stop trials, relative to situations in which recent trials have had a higher proportion of Stop trials (Figure 4). Similarly, when given a Go trial, PRO model activity is greater when the estimation of the likelihood of a Stop trial occurring is high vs. a low estimation of the likelihood of a Stop trial.

### Simulation 6: Single-unit activity

A major strength of the original PRO model is its ability to account for effects related both to the activity of ensembles of neurons (fMRI and EEG), as well as the activity of single neurons within mPFC. Here we demonstrate that, by extending the PRO model to predict events, broadly construed, it is capable of capturing additional single-unit data related to the occurrence of task-related stimuli. In earlier work (Sallet et al., 2007), single neurons in monkey ACC were observed whose activity following the presentation of an initial cue was specific to the amount of reward to be eventually received by the monkey: cues indicating small rewards activated a separate population of neurons than did cues indicating large rewards. Following a delay after the initial cue, an additional cue was presented. On the majority of trials (75%), the 2nd cue was identical to the initial cue–if the first cue indicated a small reward, the second cue did as well. On 25% of trials, however, the second cue indicated a different reward than did the first cue; if the monkey had initially been shown the small reward cue, it would now be shown the large reward cue, and vice-versa.

The authors identified two groups of neurons that appeared to code for the gain or loss of reward associated with infrequent cue switches. One group showed a large increase in activity in response to being shown a large reward cue after having been initially shown a small reward cue. These same neurons also responded (although somewhat more weakly) when the initial cue shown to the monkey was associated with the large reward. A second group of neurons showed the reverse pattern, responding strongly when a second cue indicated a small reward following an initial cue signaling a large reward, and responding more weakly when the initial cue indicated a small reward. This pattern of activity is interpreted by the authors as evidence for the hypothesis that mPFC neurons code for both unexpected events, but also specifically for reward gains and losses.

**Figure 5:**
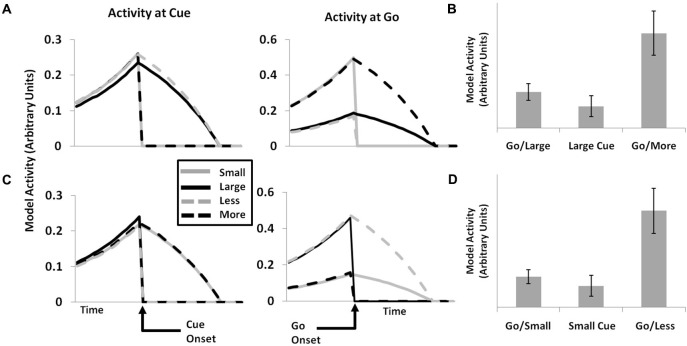
**Single-unit data (cf. Sallet et al., 2007, Figure 7)**. The activity of single units in the PRO model corresponds with data showing populations of neurons within ACC whose activity appears to code for LARGE (**A** and **B**) or SMALL (**C** and **D**) reward magnitudes. When presented with an initial cue indicating either reward magnitude (left panels), a subset of individual units remain active while the activity of other units falls to 0. Following a second GO cue (center panels), individual units appear to indicate surprising gains or losses, as would be the case when a LARGE reward is initially cued, followed by a second cue indicating a SMALL reward (top panels), and vice versa (bottom panels). Model activity is greater for GO cues than for initial cues when the reward magnitude indicated by the two cues is consistent (right panels), while activity is maximal for GO cues which are inconsistent with initial cues, either indicating a greater or lesser reward.

The notion that mPFC neurons signal discrepancies, both positive and negative, between expected and actual reward magnitudes in separate neuronal populations is broadly consistent with the theory underlying the PRO model insofar as the PRO model characterizes mPFC as a region involved in signaling deviations from expectations. The extended PRO model is able to capture the pattern of effects observed by Sallet et al. (2007), as shown in Figure 5. Rather than specifically coding for gains and losses, however, the PRO model suggests that increased activity following an unexpected second cue represents the unexpected non-occurrence of a predicted cue. This interpretation applies as well to activity observed at the presentation of the initial cue, where the prediction of the presentation of a either a cue indicating a small magnitude reward or a cue indicating a large magnitude reward is unmet.

## Discussion

In this article, we have presented an extended implementation of the PRO model of mPFC, and conducted a number of simulations showing that, using this extended framework, the model can capture an additional range of effects observed within mPFC primarily related to the detection and processing of task-related stimuli. The extended PRO model is not different from the original PRO model, in that it uses the same formal equations and parameter values. The key innovation underlying our extension to the model is conceptual—we treat stimuli and outcomes, elements of the study of behavior that have long existed at opposite ends of a trial, as being functionally equivalent in terms of their ability to serve as the basis for future predictions and to signal discrepancies between expected and actual events.

In our previous work, we noted that the PRO model offered a unifying account of mPFC activity in the context of cognitive control. The PRO model posited two main signals of prediction and comparison (i.e., prediction error) (Alexander and Brown, 2010, 2011; Brown, 2013) These functions are consistent with a variety of recent empirical results (Kennerley et al., 2011; Hayden et al., 2011a), and the prediction error signals may be a key signal that updates behavior (Hayden et al., 2011b; Kolling et al., 2012). Our recent neuroimaging findings show distinct prediction and prediction error regions within the mPFC, consistent with the PRO model (Jahn et al., 2014). In the present manuscript, we extend the earlier PRO model account to include experimental paradigms not explicitly related to response generation. Indeed, recent studies outside the purview of the original PRO model have yielded results that are readily interpretable within the framework of the extended PRO model, including findings regarding mPFC activity when monitoring the actions of others (Apps et al., 2012), during tasks focusing on predicting and detecting painful stimuli (Büchel et al., 2002; Chandrasekhar et al., 2008), or processing unexpected salient stimuli (Talmi et al., 2013). The extended PRO model here may be viewed as continually trying to build an accurate internal model of the environment. Every surprising event in turn adjusts the model to minimize future surprise. In that sense, the model is generally consistent with the theoretical principles of free energy minimization (Friston, 2010).

In addition to accounting for a new set of neural data, our present simulations provide further evidence in support of the role of mPFC in model-based RL (Dayan and Niv, 2008), implicating the mPFC in building internal models of the environment. Other studies (Gläscher et al., 2010; Ide et al., 2013) have identified signals in the brain that appear to be consistent with some form of model-based RL (as opposed to model-free RL), including signals that occur in regions that are known to interact with mPFC. Model-based RL is distinct from model-free RL in that it is concerned with learning a model of an environment, often rendered as a state-transition matrix containing the estimated probabilities of transitioning from one state to another (Simon and Daw, 2011), while model-free RL uses a scalar value signal to improve estimates of future rewards. Neurally, model-free RL is generally considered to involve primarily subcortical structures heavily innervated by dopamine neurons, including nucleus accumbens and striatum, areas that are frequently observed to respond to value and reward in decision-making tasks, and substantial research has linked DA activity in VTA to model-free RL (Cardinal and Cheung, 2005; Daw and Doya, 2006; Doya, 2007; Cohen et al., 2009).

Although it is generally accepted that complex cognitive behaviors such as planning and decision-making require that a model of the world be learned and maintained, it is still unclear what regions of the brain govern how or when a model is learned, or which regions are involved in maintaining that model. We previously noted that the vector error signal calculated by the PRO model is consistent with a state prediction error, although we note that the PRO model is not itself a model-based RL algorithm *per se*. However, it does suggest that activity in mPFC may be used as a learning signal by other brain regions that are directly involved in model maintenance (Alexander and Brown, 2011). A likely candidate in this regard is dorsolateral prefrontal cortex (PFC), a region implicated in working memory and rule representation (Wallis et al., 2001; Nee and Brown, 2013; Mian et al., 2014) and known to project reciprocally to mPFC (Barbas and Pandya, 1989). Another possible substrate of model-based prediction is the hippocampus (van der Meer and Redish, 2010). Future work should investigate how the interaction of these regions may contribute to model-based RL.

Our results show that the essential functions of the PRO model, namely that of prediction and detection of discrepancy, can account for a range of results primarily related to processing stimulus-related information. This suggests a role for mPFC in processes related to attention or attention-like processes. Previous associative (Mackintosh, 1975; Pearce and Hall, 1980), connectionist (Kruschke, 2001), and RL models (Alexander, 2007) have exploited prediction errors to drive attentional learning. One possible role of the mPFC signal may therefore involve allocating attention to relevant stimuli. We do not claim that the mPFC is the only brain region that signals prediction error though. There is evidence that other regions including the cerebellum may also signal prediction errors (Blakemore et al., 2001). An important question raised by our results concerns the distinction between the functions of orbitofrontal cortex (OFC) and mPFC. It has previously been thought that these two regions play complementary roles in decision making, with mPFC encoding action values while OFC encodes the value of stimuli (Goldstein et al., 2007; Rudebeck et al., 2008; Camille et al., 2011; Kennerley et al., 2011). The extension of the PRO model to include prediction of events in general (rather than solely predicting the consequences of actions) blurs this otherwise appealing distinction. A recent computational model (Wilson et al., 2014) interprets OFC as being involved in state representation, and thus, in conjunction with the PRO model, may provide an alternative account for the distinct, complementary roles of the two regions in model-based RL. Specifically, state representations maintained by OFC may serve as the basis for predictions generated within mPFC, while prediction errors signaled by mPFC may provide information relevant to determining task state to OFC. More generally, while the PRO model accounts for a range of data observed in mPFC, the region is highly interconnected with additional areas of the brain whose function may represent variables in the PRO model that appear to be not directly related to mPFC activity, including stimulus/state representation, the relative value of immediate options (Boorman et al., 2013), or the implementation of top-down control. Our results organize a wide range of data on the mPFC in an expanded theoretical framework, which suggests that mPFC learns to predict the outcomes of salient events in general, and provide critical constraints on the function of regions with which mPFC interacts.

A potential weakness of the current study relates to the dependence of the PRO model on consistent inter-event timing in order to converge on predictions reflecting the likelihood of observing an event. This weakness has been noted in other reports (Jahn et al., 2014), and is due to the formulation of the model based on TD learning and the temporal representation of stimuli as a tapped-delay line. The manner in which stimuli are represented through time by the brain, and how that representation informs activity in mPFC, is likely more sophisticated than the scheme implemented in the PRO model. While mPFC is known to be sensitive to violations of temporal expectancies (Yeung and Nieuwenhuis, 2009; Forster and Brown, 2011; Grinband et al., 2011), it is generally assumed that jittered delay intervals do not unduly influence BOLD activity related to underlying cognitive processes, and the use of consistent inter-event timing in our simulations reflects this assumption. However, to the extent that mPFC activity reflects deviations from temporal expectancies in addition to effects related to cognitive processes, it may be necessary to re-evaluate our current interpretations of mPFC activity in the context of a more realistic model of temporal representation.

## Conflict of interest statement

The authors declare that the research was conducted in the absence of any commercial or financial relationships that could be construed as a potential conflict of interest.

## References

[B1] Aarts E. Roelofs A. (2010). Attentional control in anterior cingulate cortex based on probabilistic cueing. J. Cogn. Neurosci. 23, 716–727 10.1162/jocn.2010.21435 20146601

[B2] Aarts E. Roelofs A. van Turennout M. (2008). Anticipatory activity in anterior cingulate cortex can be independent of conflict and error likelihood. J. Neurosci. 28, 4671–4678 10.1523/jneurosci.4400-07.2008 18448644 PMC6670453

[B3] Alexander W. H. (2007). Shifting attention using a temporal difference prediction error and high-dimensional input. Adapt. Behav. 15, 121–133 10.1177/1059712307078663

[B4] Alexander W. H. Brown J. W. (2010). Computational models of performance monitoring and cognitive control. Top. Cogn. Sci. 2, 658–677 10.1111/j.1756-8765.2010.01085.x 21359126 PMC3044326

[B5] Alexander W. H. Brown J. W. (2011). Medial prefrontal cortex as an action-outcome predictor. Nat. Neurosci. 14, 1338–1344 10.1038/nn.2921 21926982 PMC3183374

[B6] Amador N. Schlag-Rey M. Schlag J. (2000). Reward-predicting and reward-detecting neuronal activity in the primate supplementary eye field. J. Neurophysiol. 84, 2166–2170 11024104 10.1152/jn.2000.84.4.2166

[B7] Amiez C. Joseph J. P. Procyk E. (2006). Reward encoding in the monkey anterior cingulate cortex. Cereb. Cortex 16, 1040–1055 10.1093/cercor/bhj046 16207931 PMC1913662

[B8] Apps M. A. J. Balsters J. H. Ramnani N. (2012). The anterior cingulate cortex: monitoring the outcomes of others’ decisions. Soc. Neurosci. 7, 424–435 10.1080/17470919.2011.638799 22114875

[B67] Barbas H. Pandya D. N. (1989). Architecture and intrinsic connections of the prefrontal cortex in the rhesus monkey. J. Comp. Neurol. 286, 353–375 10.1002/cne.902860306 2768563

[B9] Behrens T. E. Woolrich M. W. Walton M. E. Rushworth M. F. (2007). Learning the value of information in an uncertain world. Nat. Neurosci. 10, 1214–1221 10.1038/nn1954 17676057

[B10] Blais C. Bunge S. (2010). Behavioral and neural evidence for item-specific performance monitoring. J. Cogn. Neurosci. 22, 2758–2767 10.1162/jocn.2009.21365 19925177

[B11] Blakemore S.-J. Frith C. Wolpert D. M. (2001). The cerebellum is involved in predicting the sensory consequences of action. [Miscellaneous Article]. Neuroreport 12, 1879–1884 10.1097/00001756-200107030-00023 11435916

[B12] Boorman E. D. Rushworth M. F. Behrens T. E. (2013). Ventromedial prefrontal and anterior cingulate cortex adopt choice and default reference frames during sequential multialternative choice. J. Neurosci. 33, 2242–2253 10.1523/jneurosci.3022-12.2013 23392656 PMC3743024

[B13] Botvinick M. M. Braver T. S. Barch D. M. Carter C. S. Cohen J. C. (2001). Conflict monitoring and cognitive control. Psychol. Rev. 108, 624–652 10.1037/0033-295x.108.3.624 11488380

[B14] Brown J. W. (2013). Beyond conflict monitoring cognitive control and the neural basis of thinking before you act. Curr. Dir. Psychol. Sci. 22, 179–185 10.1177/0963721412470685 PMC4210858 25360064

[B15] Brown J. W. Braver T. S. (2005). Learned predictions of error likelihood in the anterior cingulate cortex. Science 307, 1118–1121 10.1126/science.1105783 15718473

[B16] Bryden D. W. Johnson E. E. Tobia S. C. Kashtelyan V. Roesch M. R. (2011). Attention for learning signals in anterior cingulate cortex. J. Neurosci. 31, 18266–18274 10.1523/JNEUROSCI.4715-11.2011 22171031 PMC3285822

[B17] Büchel C. Bornhövd K. Quante M. Glauche V. Bromm B. Weiller C. (2002). Dissociable neural responses related to pain intensity, stimulus intensity and stimulus awareness within the anterior cingulate cortex: a parametric single-trial laser functional magnetic resonance imaging study. J. Neurosci. 22, 970–976 11826125 10.1523/JNEUROSCI.22-03-00970.2002 PMC6758484

[B18] Bugg J. M. Jacoby L. L. Toth J. P. (2008). Multiple levels of control in the Stroop task. Mem. Cognit. 36, 1484–1494 10.3758/MC.36.8.1484 19015507 PMC2682765

[B19] Camille N. Tsuchida A. Fellows L. K. (2011). Double dissociation of stimulus-value and action-value learning in humans with orbitofrontal or anterior cingulate cortex damage. J. Neurosci. 31, 15048–15052 10.1523/JNEUROSCI.3164-11.2011 22016538 PMC6623552

[B20] Cardinal R. N. Cheung T. H. C. (2005). Nucleus accumbens core lesions retard instrumental learning and performance with delayed reinforcement in the rat. BMC Neurosci. 6:9 10.1186/1471-2202-6-9 15691387 PMC549214

[B21] Carter C. S. Braver T. S. Barch D. M. Botvinick M. M. Noll D. C. Cohen J. D. (1998). Anterior cingulate cortex, error detection and the online monitoring of performance. Science 280, 747–749 10.1126/science.280.5364.747 9563953

[B22] Carter C. S. Macdonald A. M. Botvinick M. Ross L. L. Stenger V. A. Noll D. (2000). Parsing executive processes: strategic vs. evaluative functions of the anterior cingulate cortex. Proc. Natl. Acad. Sci. U S A 97, 1944–1948 10.1073/pnas.97.4.1944 10677559 PMC26541

[B23] Chandrasekhar P. V. S. Capra C. M. Moore S. Noussair C. Berns G. S. (2008). Neurobiological regret and rejoice functions for aversive outcomes. Neuroimage 39, 1472–1484 10.1016/j.neuroimage.2007.10.027 18042401 PMC2265597

[B24] Cohen M. X. Axmacher N. Lenartz D. Elger C. E. Sturm V. Schlaepfer T. E. (2009). Neuroelectric signatures of reward learning and decision-making in the human nucleus accumbens. Neuropsychopharmacology 34, 1649–1658 10.1038/npp.2008.222 19092783

[B25] Crottaz-Herbette S. Menon V. (2006). Where and when the anterior cingulate cortex modulates attentional response: combined fMRI and ERP evidence. J. Cogn. Neurosci. 18, 766–780 10.1162/jocn.2006.18.5.766 16768376

[B26] Daw N. D. Doya K. (2006). The computational neurobiology of learning and reward. Curr. Opin. Neurobiol. 16, 199–204 10.1016/j.conb.2006.03.006 16563737

[B27] Dayan P. Niv Y. (2008). Reinforcement learning: the good, the bad and the ugly. Curr. Opin. Neurobiol. 18, 185–196 10.1016/j.conb.2008.08.003 18708140

[B28] Dien J. Spencer K. M. Donchin E. (2003). Localization of the event-related potential novelty response as defined by principal components analysis. Brain Res. Cogn. Brain Res. 17, 637–650 10.1016/s0926-6410(03)00188-5 14561451

[B29] Downar J. Crawley A. P. Mikulis D. J. Davis K. D. (2002). A cortical network sensitive to stimulus salience in a neutral behavioral context across multiple sensory modalities. J. Neurophysiol. 87, 615–620 11784775 10.1152/jn.00636.2001

[B30] Doya K. (2007). Reinforcement learning: computational theory and biological mechanisms. HFSP J. 1, 30–40 10.2976/1.2732246/10.2976/1 19404458 PMC2645553

[B31] Eriksen C. W. (1995). The flankers task and response competition: a useful tool for investigating a variety of cognitive problems. Vis. Cogn. 2, 101–118 10.1080/13506289508401726

[B32] Falkenstein M. Hohnsbein J. Hoorman J. Blanke L. (1991). Effects of crossmodal divided attention on late ERP components: II. Error processing in choice reaction tasks. Electroencephalogr. Clin. Neurophysiol. 78, 447–455 10.1016/0013-4694(91)90062-9 1712280

[B33] Forster S. E. Brown J. W. (2011). Medial prefrontal cortex predicts and evaluates the timing of action outcomes. Neuroimage 55, 253–265 10.1016/j.neuroimage.2010.11.035 21094259 PMC3031730

[B34] Friston K. (2010). The free-energy principle: a unified brain theory? Nat. Rev. Neurosci. 11, 127–138 10.1038/nrn2787 20068583

[B35] Gläscher J. Daw N. Dayan P. O’Doherty J. P. (2010). States versus rewards: dissociable neural prediction error signals underlying model-based and model-free reinforcement learning. Neuron 66, 585–595 10.1016/j.neuron.2010.04.016 20510862 PMC2895323

[B36] Goldstein R. Z. Tomasi D. Rajaram S. Cottone L. A. Zhang L. Maloney T. (2007). Role of the anterior cingulate and medial orbitofrontal cortex in processing drug cues in cocaine addiction. Neuroscience 144, 1153–1159 10.1016/j.neuroscience.2006.11.024 17197102 PMC1852512

[B37] Grinband J. Savitskaya J. Wager T. D. Teichert T. Ferrera V. P. Hirsch J. (2011). The dorsal medial frontal cortex is sensitive to time on task, not response conflict or error likelihood. Neuroimage 57, 303–311 10.1016/j.neuroimage.2010.12.027 21168515 PMC3114292

[B38] Hayden B. Y. Heilbronner S. R. Pearson J. M. Platt M. L. (2011a). Surprise signals in anterior cingulate cortex: neuronal encoding of unsigned reward prediction errors driving adjustment in behavior. J. Neurosci. 31, 4178–4187 10.1523/JNEUROSCI.4652-10.2011 21411658 PMC3070460

[B39] Hayden B. Y. Pearson J. M. Platt M. L. (2011b). Neuronal basis of sequential foraging decisions in a patchy environment. Nat. Neurosci. 14, 933–939 10.1038/nn.2856 21642973 PMC3553855

[B40] Holroyd C. B. Coles M. G. (2002). The neural basis of human error processing: reinforcement learning, dopamine and the error-related negativity. Psychol. Rev. 109, 679–709 10.1037//0033-295x.109.4.679 12374324

[B41] Ide J. S. Shenoy P. Yu A. J. Li C. R. (2013). Bayesian prediction and evaluation in the anterior cingulate cortex. J. Neurosci. 33, 2039–2047 10.1523/JNEUROSCI.2201-12.2013 23365241 PMC3711643

[B42] Jahn A. Nee D. E. Alexander W. H. Brown J. W. (2014). Distinct regions of anterior cingulate cortex signal prediction and outcome evaluation. Neuroimage 95, 80–89 10.1016/j.neuroimage.2014.03.050 24667454 PMC4077597

[B44] Jessup R. K. Busemeyer J. R. Brown J. W. (2010). Error effects in anterior cingulate cortex reverse when error likelihood is high. J. Neurosci. 30, 3467–3472 10.1523/JNEUROSCI.4130-09.2010 20203206 PMC2841347

[B45] Kennerley S. W. Behrens T. E. J. Wallis J. D. (2011). Double dissociation of value computations in orbitofrontal and anterior cingulate neurons. Nat. Neurosci. 14, 1581–1589 10.1038/nn.2961 22037498 PMC3225689

[B46] Kolling N. Behrens T. E. J. Mars R. B. Rushworth M. F. S. (2012). Neural mechanisms of foraging. Science 336, 95–98 10.1126/science.1216930 22491854 PMC3440844

[B47] Koyama T. Kato K. Tanaka Y. Z. Mikami A. (2001). Anterior cingulate activity during pain-avoidance and reward tasks in monkeys. Neurosci. Res. 39, 421–430 10.1016/s0168-0102(01)00197-3 11274741

[B48] Kruschke J. K. (2001). Toward a unified model of attention in associative learning. J. Math. Psychol. 45, 812–863 10.1006/jmps.2000.1354

[B49] Litt A. Plassmann H. Shiv B. Rangel A. (2011). Dissociating valuation and saliency signals during decision-making. Cereb. Cortex 21, 95–102 10.1093/cercor/bhq065 20444840

[B50] Mackintosh N. (1975). A theory of attention: variations in the associability of stimuli with reinforcement. Psychol. Rev. 82, 276–298 10.1037/h0076778

[B51] Matsumoto M. Matsumoto K. Abe H. Tanaka K. (2007). Medial prefrontal cell activity signaling prediction errors of action values. Nat. Neurosci. 10, 647–656 10.1038/nn1890 17450137

[B52] Mian M. K. Sheth S. A. Patel S. R. Spiliopoulos K. Eskandar E. N. Williams Z. M. (2014). Encoding of rules by neurons in the human dorsolateral prefrontal cortex. Cereb. Cortex 24, 807–816 10.1093/cercor/bhs361 23172774 PMC3920771

[B53] Nee D. E. Brown J. W. (2013). Dissociable frontal-striatal and frontal-parietal networks involved in updating hierarchical contexts in working memory. Cereb. Cortex 23, 2146–2158 10.1093/cercor/bhs194 22798339 PMC3841420

[B54] Pearce J. M. Hall G. (1980). A model for pavlovian learning: variations in the effectiveness of conditioned but not of unconditioned stimuli. Psychol. Rev. 87, 532–552 10.1037/0033-295x.87.6.532 7443916

[B55] Rudebeck P. H. Behrens T. E. Kennerley S. W. Baxter M. G. Buckley M. J. Walton M. E. (2008). Frontal cortex subregions play distinct roles in choices between actions and stimuli. J. Neurosci. 28, 13775–13785 10.1523/jneurosci.3541-08.2008 19091968 PMC6671924

[B56] Sallet J. Quilodran R. Rothé M. Vezoli J. Joseph J.-P. Procyk E. (2007). Expectations, gains, and losses in the anterior cingulate cortex. Cogn. Affect. Behav. Neurosci. 7, 327–336 10.3758/cabn.7.4.327 18189006 PMC2271114

[B57] Shima K. Tanji J. (1998). Role of cingulate motor area cells in voluntary movement selection based on reward. Science 282, 1335–1338 10.1126/science.282.5392.1335 9812901

[B68] Simon D. A. Daw N. D. (2011). Neural correlates of forward planning in a spatial decision task in humans. J. Neurosci. 31, 5526–5539 10.1523/JNEUROSCI.4647-10.2011 21471389 PMC3108440

[B58] Stroop J. R. (1935). Studies of interference in serial verbal reactions. J. Exp. Psychol. 18, 643–662 10.1037/h0054651

[B59] Sutton R. S. (1988). Learning to predict by the methods of temporal difference. Mach. Learn. 3, 9–44 10.1007/bf00115009

[B60] Talmi D. Atkinson R. El-Deredy W. (2013). The feedback-related negativity signals salience prediction errors, not reward prediction errors. J. Neurosci. 33, 8264–8269 10.1523/jneurosci.5695-12.2013 23658166 PMC6619637

[B61] Vachon F. Hughes R. W. Jones D. M. (2012). Broken expectations: violation of expectancies, not novelty, captures auditory attention. J. Exp. Psychol. Learn. Mem. Cogn. 38, 164–177 10.1037/a0025054 21895389

[B62] van der Meer M. A. Redish A. D. (2010). Expectancies in decision making, reinforcement learning and ventral striatum. Front. Neurosci. 4:6 10.3389/neuro.01.006.2010 21221409 PMC2891485

[B63] Wallis J. D. Anderson K. C. Miller E. K. (2001). Single neurons in prefrontal cortex encode abstract rules. Nature 411, 953–956 10.1038/35082081 11418860

[B64] Wessel J. R. Danielmeier C. Morton J. B. Ullsperger M. (2012). Surprise and error: common neuronal architecture for the processing of errors and novelty. J. Neurosci. 32, 7528–7537 10.1523/jneurosci.6352-11.2012 22649231 PMC6703591

[B69] Wilson R. C. Takahashi Y. K. Schoenbaum G. Niv Y. (2014). Orbitofrontal cortex as a cognitive map of task space. Neuron 81, 267–279 10.1016/j.neuron.2013.11.005 24462094 PMC4001869

[B65] Yeung N. Nieuwenhuis S. (2009). Dissociating response conflict and error likelihood in anterior cingulate cortex. J. Neurosci. 29, 14506–14510 10.1523/jneurosci.3615-09.2009 19923284 PMC2831178

[B66] Yu A. J. Dayan P. Cohen J. D. (2009). Dynamics of attentional selection under conflict: toward a rational Bayesian account. J. Exp. Psychol. Hum. Percept. Perform. 35, 700–717 10.1037/a0013553 19485686 PMC3432507

